# Characterization of High-Temperature Superconductor Bulks for Electrical Machine Application

**DOI:** 10.3390/ma14071636

**Published:** 2021-03-26

**Authors:** Bruno Douine, Kevin Berger, Nickolay Ivanov

**Affiliations:** 1 Groupe de Recherche en Energie Electrique de Nancy (GREEN), Université de Lorraine, 54506 Vandoeuvre-lès-Nancy, France; kevin.berger@univ-lorraine.fr; 2 Moscow Aviation Institute (MAI), 125993 Moscow, Russia; n.s.ivanov88@gmail.com

**Keywords:** high-temperature superconductor, characterization, electrical motor

## Abstract

High-temperature superconducting (HTS) bulks can be used in electrical applications. Experimental characterization of large-size HTS bulks is a tricky issue. The relevant parameters for their application were directly measured in this study. This paper has three main aims. Firstly, features of YBaCuO bulks are presented. Secondly, an electrical motor application is developed using magnetic field shielding and trapping. Thirdly, the HTS bulks are characterized. Several classical methods were used, which are mainly magnetic methods only available for small samples. The complete penetration magnetic field and the critical current density were found to be the main parameters relevant for applications. An innovative entire HTS bulk characterization method is presented. This characterization method is useful for end users and engineers to better implement HTS bulks.

## 1. Introduction

High-temperature superconducting (HTS) bulks can be used in electrical applications as magnets or magnetic shields. Indeed, they have a high critical current density *J_C_* and can be magnetized to very high magnetic flux densities. The main applications of HTS bulks are levitation, cryo-magnets, electrical motors, and magnetic gears.

It is necessary to characterize this HTS bulks to optimally use them. This means the determination of the critical current density *J_C_*. Different classical methods have been used for small samples [1,2,3,4,5,6,7,8,9,10,11,12,13,14,15,16,17,18,19,20,21]. The goal of these classical methods is mainly to obtain the dependence of *J_C_* on the magnetic field B and temperature of the HTS material. Different characterization methods exist, such as magnetization methods, measurements of susceptibility, or measurements of levitation force. In these classical characterization methods, the relationship between measurements and *J_C_* is not direct and, thus, these are called indirect methods. On another hand, Mawatari [9,10] proposed using an electric characterization method to characterize HTS bulks, by cutting a strip in the HTS bulk. This allows directly determining *J_C_*, but it modifies the properties of the HTS sample in comparison with the entire HTS bulk. The main issue with these characterization methods is that there are valid only for small samples cut into entire HTS bulks. This is sufficient for material scientists and it allows comparing HTS materials, but it is not useful for applications. End users of HTS bulks, such as engineers or electrical engineering researchers, need characterization of the entire HTS bulks to optimally use them in applications. Hence, for real HTS bulks used in electrical applications, other methods are necessary, allowing characterization of the entire HTS bulks without cutting. Recently, the CAN company [8], building and selling HTS YBaCuO bulks, proposed characterizing different samples of HTS material placed at different regions in the YBaCuO bulk to better characterize their products. This proves that companies need a characterization method for entire HTS bulk. This kind of characterization method is at the boundary between material scientists, companies, and engineers. Previously, Vanderbemden [17] used pick up coils and Hall probes to characterize entire HTS bulks with a magnetization cycle. In the last few last years, we proposed a new direct magnetic determination method for *J_C_* and *n*-value. It consists of the determination of the complete penetration magnetic field *B_P_* and a deduction of the critical current density *J_C_*. This new method is original, quick, and direct for determining the critical current density *Jc* and *n*-value in bulk superconductors from magnetic field diffusion measurements [18,19,20,21]. Experimental characterization of large-size samples is a tricky issue. This process is considered an “engineering characterization” of the HTS bulks used in application. Thus, the relevant parameters, such as the complete penetration magnetic field, for applications should be directly measured.

This paper has three main aims. Firstly, features of YBaCuO bulks are presented and compared with respect to applications. Secondly, an HTS electrical motor application is presented. Lastly, the innovative HTS bulk characterization method is described. 

## 2. High-Temperature Superconducting Bulks

### 2.1. Composition of HTS Bulks

HTS bulks are composed of copper-oxide layers and a rare earth element as Yttrium [22]. The superconductivity exists in the *a*–*b* plane of the copper-oxide layer. In the *c*-direction, perpendicular to the *a*–*b* plane, the electric conductivity is close to zero. Thus, HTS materials are clearly anisotropic.

**Figure 1:**
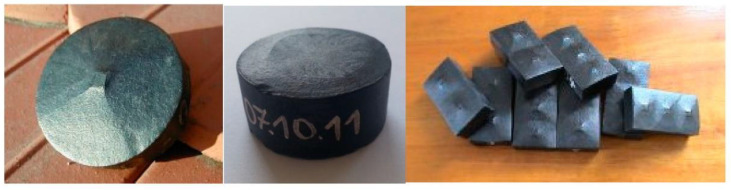
Mono crystal high-temperature superconducting (HTS) bulks from ATZ company.

The HTS bulks used for applications as motors or super magnets are mono crystals. More than 20 years ago, top-seeded melt growth (TSMG) was established, which allowed building large, single-grain HTS bulks [23]. The process of HTS bulk fabrication is the growing of one mono crystal from a seed at the center of the HTS bulk. Figure 1 shows the seed at the center of HTS bulks. These materials have significant potential for application as high-field permanent magnets. The performance of these magnets at 77 K is limited by the critical current carrying capacity of the bulk superconductor. The values of *J_C_* are more than 100 A/mm^2^ at 77 K, and the size can be more than 5 cm diameter (Figure 1) with different shapes. 

### 2.2. Critical Quantities

As a superconducting material, HTS bulks have three critical quantities: critical temperature *T_C_* (92 K for YBaCuO), critical current density *J_C_*, and critical magnetic field *H_C_*. Because HTS materials are used at high temperature (T > 30 K), there is another important magnetic field, called the irreversibility magnetic field H*, between *H_C_*_1_, where the penetration of the magnetic field begins, and *H_C_*_2_, where the superconductivity disappears. If the magnetic field inside the HTS material is higher than this irreversibility magnetic field, the critical current density drops to zero. Thus, H* is the most important magnetic field parameter to know for electric applications as motors, because, in this case, a high electric current density and high magnetic field are simultaneously necessary to improve motor capability and efficiency.

## 3. HTS Bulk Application in Electrical Machines

HTS bulk can be used as a magnetic shield or permanent magnet in superconducting electrical machines.

### 3.1. Magnetic Flux Concentration Machines from GREEN Lab

GREEN lab in France designed, built, and tested many HTS machines using a magnetic shield producing a magnetic field concentration.

**Figure 2:**
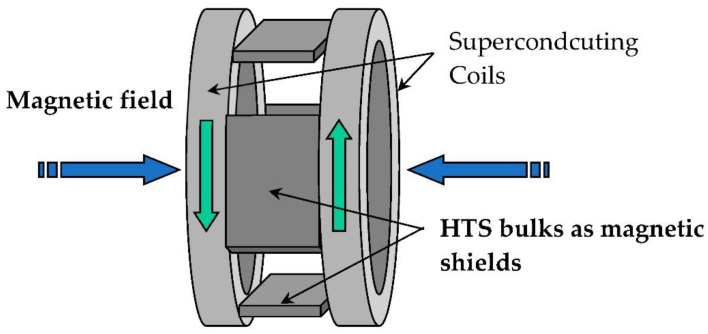
Inductor of the motor with HTS bulk magnetic flux concentration.

**Figure 3:**
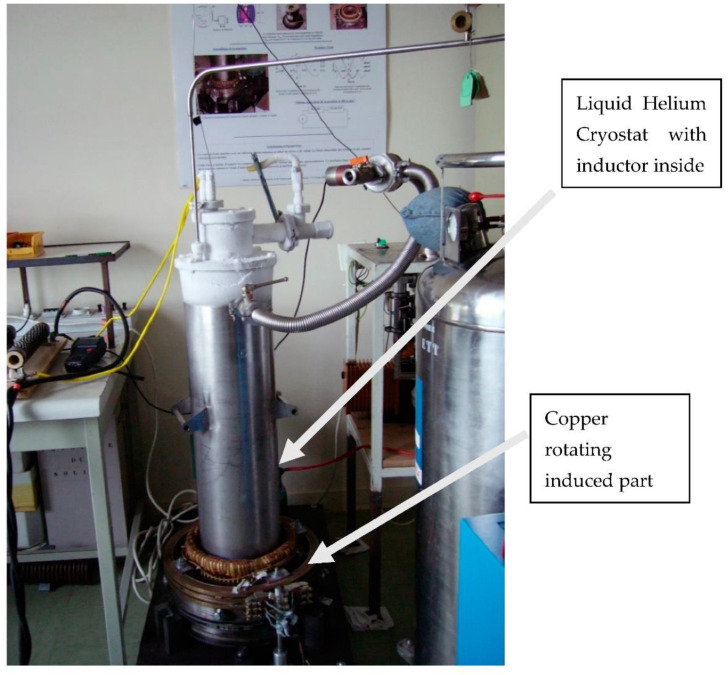
HTS bulk magnetic flux concentration inductor motor.

The inductor of the first prototype built in GREEN was originally presented in [24]. It used the high capacity of two superconducting coils (Figure 2) to produce a high magnetic field without iron (Figure 2) and the magnetic shielding of HTS bulks. The two superconducting coils create two magnetic fields in opposition, which create a high radial magnetic field between them. The HTS bulks used as magnetic field shields create spatial variation of the magnetic field along the perimeter of the inductor. This spatial variation can be used to generate alternating current (AC) voltage in induced part coils or to produce torque between this inductor and induced part. HTS materials enable reaching levels of magnetic flux density much higher than those obtained using classic magnetic materials, This prototype (Figure 3) was built and tested on the basis of this principle [25]. The coils of the induced part were made with copper because only the inductor was cooled down at the temperature of liquid helium. Liquid helium was chosen as the cooling system because it is a simple solution to cool down the NbTi coils producing a high magnetic field, which ensures that the HTS bulks perfectly shield this high magnetic field. Actually, a higher temperature reduces the magnetic shielding of the HTS bulks because the penetration depth of the magnetic field inside the HTS bulk depends on the critical current density *J_C_*, which decreases when temperature increases.

**Figure 4:**
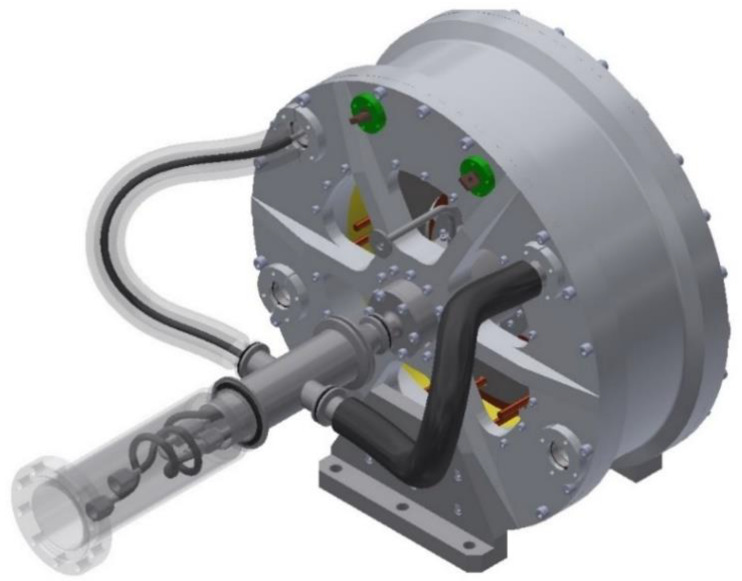
HTS motor from GREEN and SAFRAN for aeronautic applications.

Following this first machine, a collaboration began a few years ago between SAFRAN and GREEN to construct an HTS motor for aeronautic applications (Figure 4). 

**Figure 5:**
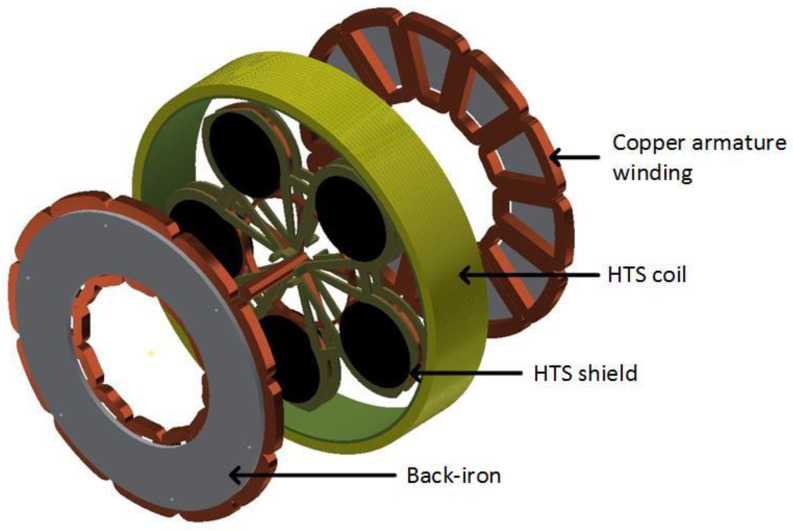
HTS coils producing an axial magnetic field, with HTS bulks as a magnetic shield and armature windings.

In this second magnetic flux concentration motor [26], the HTS coil produces a high axial magnetic field, and five HTS pellets, which rotate, shield the magnetic field and concentrate the magnetic field between them. The armature is made with copper winding at room temperature, because the AC losses would be too high at low temperature (Figure 5). This motor is under patent, thus, further details are classified.

### 3.2. HTS Permanent Magnet Machine

**Figure 6:**
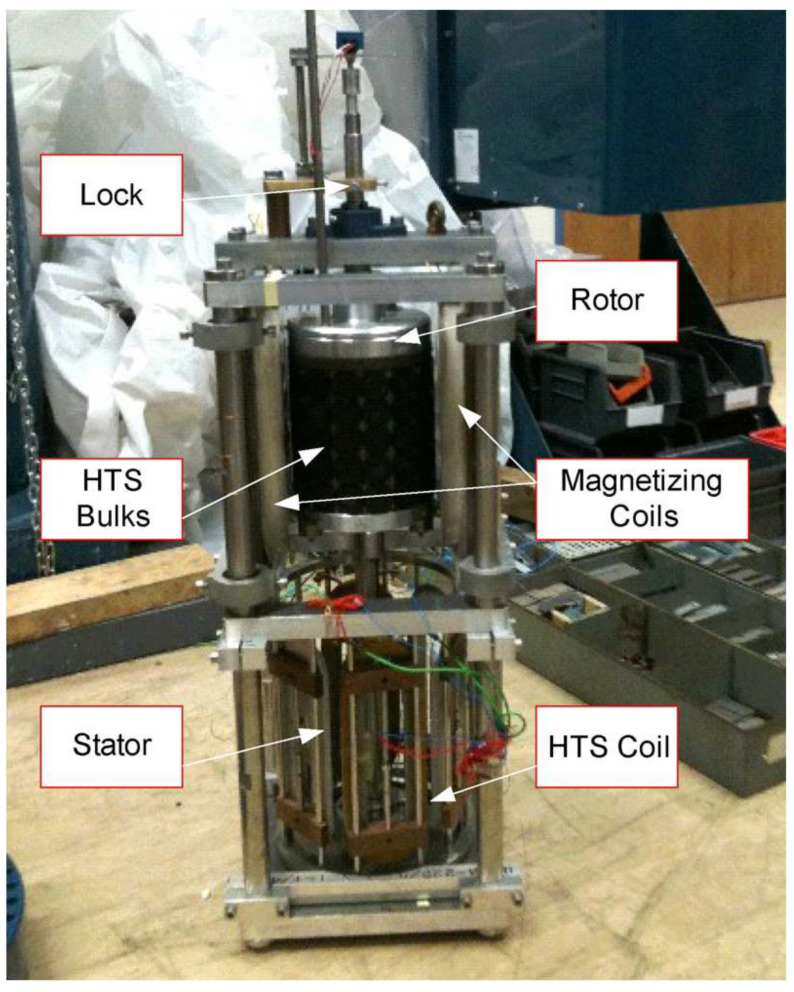
HTS bulk motor designed and built by Dr Coombs et al. [34].

The HTS YBaCuO bulk, submitted to a magnetic field and then cooled down, traps the magnetic field. This method is called zero field cooling (ZFC). It is possible to obtain 4 T on the surface of a YBaCuO bulk at 77 K (liquid nitrogen) and up to 17 T at 29 K [27] and 17.9 T at 26 K [23]. The ZFC method takes time and a huge superconducting coil. Thus, magnetization of HTS bulks is not suitable for motor applications. A suitable magnetization method exists called pulse field magnetization (PFM). In this method, the HTS YBaCuO bulk is cooled down and then submitted to magnetic field pulses [28,29,30]. It traps less of the magnetic field than the ZFC method but it requires only a small copper magnetization coil. It allows the production of prototypes where HTS bulks on a rotor are magnetized and used as permanent magnets. In Japan, Professor Izumi and his team have been working on this topic for many years, and they have built several prototypes [31,32,33]. In England, Dr. Coombs has also built a prototype [34] (Figure 6).

One of the issues with this kind of HTS motor is the choice of whether to magnetize the HTS bulks inside the machine (in situ) or outside of the machine (ex situ). The prototypes already made used ex situ magnetization because it is the simplest option in terms of magnetization. However, many teams [28,29,30,31] are working on in situ magnetization for future industrial applications because it should be simpler for the end user.

**Figure 7:**
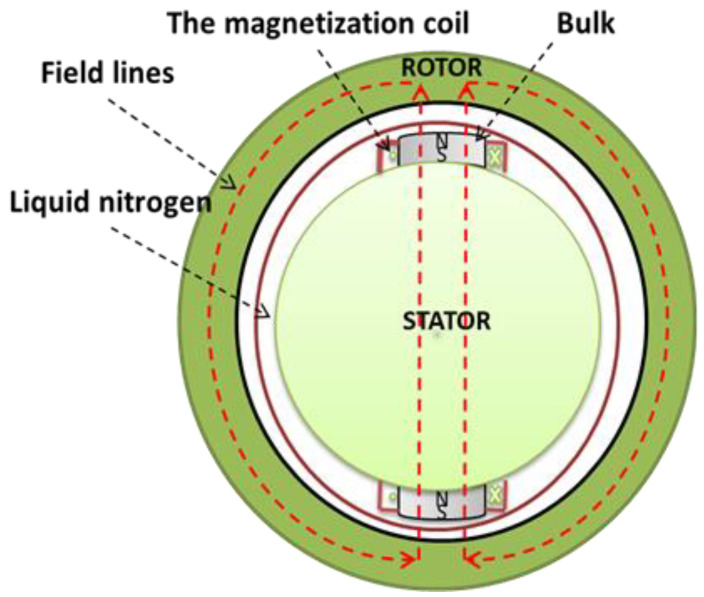
Simplified magnetic circuit of HTS bulk motor.

Trapping the magnetic field in the HTS bulk using the PFM method depends on the critical current density *J_C_*, the size of the HTS bulk, and the form of the applied magnetic field pulse [28,29,30]. In a synchronous HTS motor, the HTS bulks replace permanent magnets. Figure 7 presents a simplified model of the magnetic circuit with an HTS bulk plug on the rotor of an electrical machine. Field lines are in the air, iron, and superconducting bulk. GREEN lab studied the magnetization of HTS bulk in an iron core (Figure 7), and they concluded that the trapped magnetic field in the HTS bulk increased and the current of the optimal pulse decreased. Therefore, iron around the HTS bulk improved the magnetization process [29,30]. 

## 4. Characterization of HTS Bulks

In this section, the HTS bulk characterization method developed by us is presented; however, the complete penetration magnetic field is firstly defined.

### 4.1. Definition of the Complete Penetration Magnetic Field B_P_

The most used definition of the complete penetration magnetic field *B_P_* is generally linked to the Bean model [1]. In this model, the current density is assumed equal to the critical current density *J_C_* or null in the superconducting material. With this model, the penetration depth *d_P_* of the magnetic field in a superconducting infinite cylinder submitted to an axial applied magnetic field *Ba* is calculated as

(1) $$d_{P}=\frac{B_{a}}{\mu_{0}J_{C}}.$$

Therefore, if the magnetic field reaches the center of the infinite cylinder, where *d_P_* = *R* is the radius of the cylinder, the complete penetration magnetic field is defined by

(2) $$B_{P}=\mu_{0}J_{C}R.$$

For electrical motor applications using HTS bulk, the penetration depth *d_P_*, related to *J_C_*, allows determining the capability of magnetic shielding. On the other hand, the complete penetration magnetic field *B_P_* allows determining the capability of magnetic field trapping of HTS bulks.

The shape of *Ba*(*t*) does not matter with the Bean model; hence, we applied the following equation:

(3) $$B_{a}\left(t\right)=V_{b}\cdot t.$$

**Figure 8:**
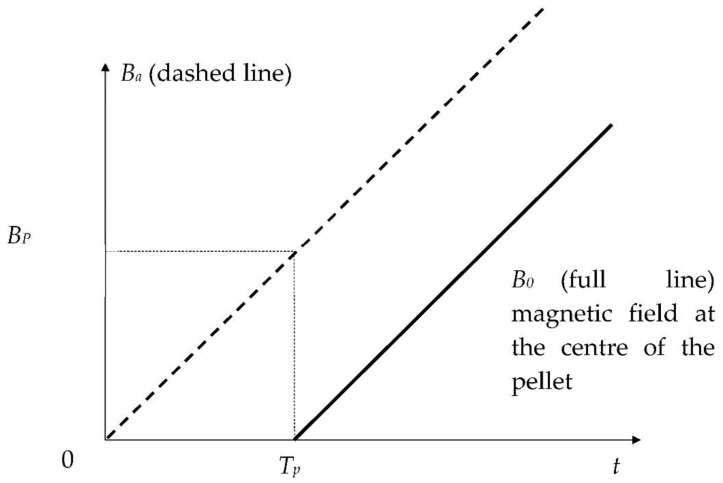
Applied magnetic field *Ba*(*t*) and magnetic induction at the center of the cylindrical HTS bulk *Bo*(*t*).

At the center of the HTS bulk, the magnetic field *Bo* begins to increase with delay time *T_P_* when *Ba* reaches the complete magnetic field *B_P_* (Figure 8).

**Figure 9:**
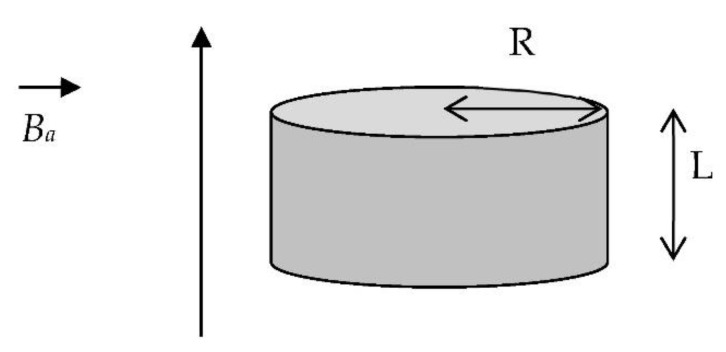
A cylindrical HTS bulk with its axis parallel to the applied magnetic field.

For a cylindrical HTS bulk of length L and radius R, submitted to an applied magnetic field *Ba*(*t*) (Figure 9), the following explicit expression of the penetration magnetic field *B_P_* can be calculated using the Bean model [32]:

(4) $$B_{P}=\frac{\mu_{0}J_{C}\cdot L}{4}\cdot ln\left(\frac{\sqrt{R^{2}+{\left(\frac{L}{2}\right)}^{2}}+R}{\sqrt{R^{2}+{\left(\frac{L}{2}\right)}^{2}}-R}\right),$$

where *L* is the length of the cylinder and *R* is its radius. Equation (2) is the equivalent of the *B_p_* formula given by different authors [18,19,20,21].

Thus, from *B_P_* and the geometric parameters of the cylindrical HTS pellet, *J_C_* can be deduced with the help of Equation (4).

In the case of HTS used at “high temperatures” (typically above 50–60 K), a power law model (PLM) better represents the E(J) characteristic of the materials than the Bean model. The PLM is typically written as

(5) $$E=E_{C}{\left(\frac{J}{J_{C}}\right)}^{n}=\frac{E_{C}.}{J_{C}}{\left(\frac{J}{J_{C}}\right)}^{n-1}J.$$

The determination of *B_P_*, *J_C_*, and *n*-value of HTS bulks can be done by measurement and numerical simulation of magnetic field penetration [19]. The influence of the rise rate of the applied magnetic field *V_b_* changes the distribution of the magnetic field and current density according to the power law (Equation (5)) instead of the Bean model [17]. Thus, *B_P_* depends on the rise rate *V_b_* as follows:

(6) $$B_{P}=\frac{\mu_{0}J_{C}\cdot L}{4}\cdot ln\left(\frac{\sqrt{R^{2}+{\left(\frac{L}{2}\right)}^{2}}+R}{\sqrt{R^{2}+{\left(\frac{L}{2}\right)}^{2}}-R}\right)\left(1+\frac{\alpha lnV_{b}+\beta }{n}\right),$$

where *α* = 1.2 and *β* = 3.4 for a typical HTS bulk size.

### 4.2. Presentation of the Characterization Method

**Figure 10:**
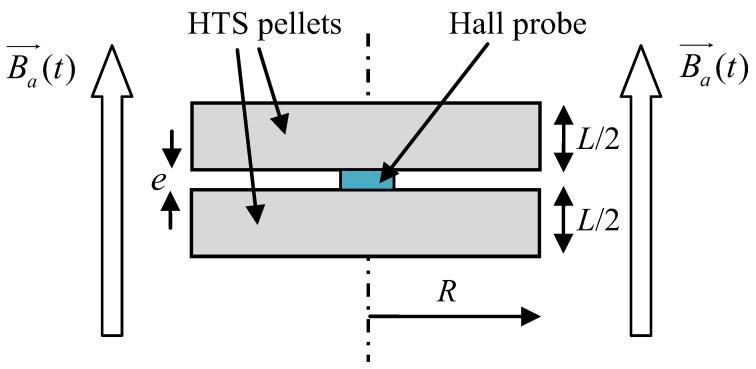
Hall probe location between two HTS bulks.

**Figure 11:**
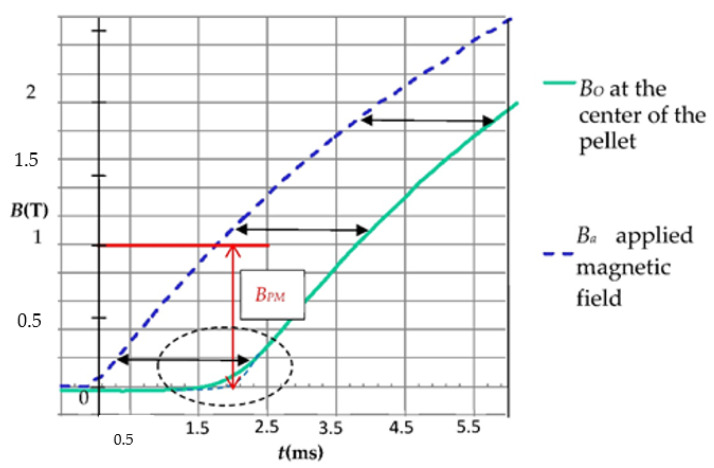
Applied magnetic field *Ba* and measured magnetic field *Bo* at the center of the HTS bulk versus time.

In this method, the HTS sample is submitted to an axial applied magnetic field *Ba*(*t*) (Figure 8). *Ba*(*t*) is a linear function of time. In our method, the complete penetration magnetic field is detected. To measure *B_P_* [18,19,20], an axial Hall probe, placed between two identical cylindrical HTS bulks (Figure 10) at their center, detects the penetration of the magnetic field. Theoretically, these two pellets are considered as one unique pellet. Therefore, the influence of the Hall probe thickness e is neglected. At the position of the Hall probe, the magnetic field has only an axial component; thus, the magnetic field can be well measured with this axial Hall probe. In our experiment (Figure 11), the applied magnetic field *Ba*(*t*) had a constant rise rate *V_b_* of ~660 T/s. As shown in the red region of Figure 11, the beginning of *B*_0_(*t*) does not have exactly the same shape as the beginning of *Ba*(*t*). This is due to the fact that this is not a single HTS bulk but two bulks separated by the Hall probe thickness. The influence of the thickness of the Hall probe e is presented below. The determination of the penetration time *T_P_* is done as follows: the measured magnetic field curve at the pellet center is modified around *T_P_* to be a copy of applied magnetic field *Ba*(*t*) (dotted line in Figure 11). Throughout time, the difference between curves is constant and equal to *T_P_*. *T_P_* is determined, and then *B_P_* can be deduced from the curves.

The influence of Hall probe thickness e (Figure 10) was calculated with the help of a numerical simulation [19]. The thickness of the Hall probe e was around 0.5 mm. The relationship between the measured complete penetration magnetic field *B_PM_* and the theoretical *B_P_* from (Equation (6)) is as follows:

(7) $$\frac{B_{PM}}{B_{P}}\left(e=0.5\;\mathrm{mm}\right)=0.031ln\left(L\right)+0.82.$$

For the two YBaCuO bulks presented in Figure 11, the measured *B_PM_* was around 1 T (red arrow). *B_P_* was deduced from *B_PM_* using Equation (7) and knowing *e* and *L*. *J_C_* was deduced from *B_P_* with Equation (6) and with *n* taken as equal to 50, giving a result of ~110 A/mm^2^.

**Figure 12:**
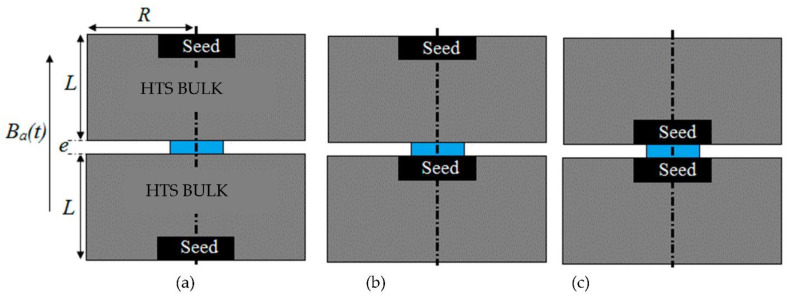
Different positions of the HTS bulk seeds (in black) in relation to the Hall probe (in blue). For case (**a**) the two seeds far from the Hall probe, case (**b**) one seed far from the Hall probe and one seed close to the Hall probe and case (**c**) the two seeds close to the Hall probe.

**Figure 13:**
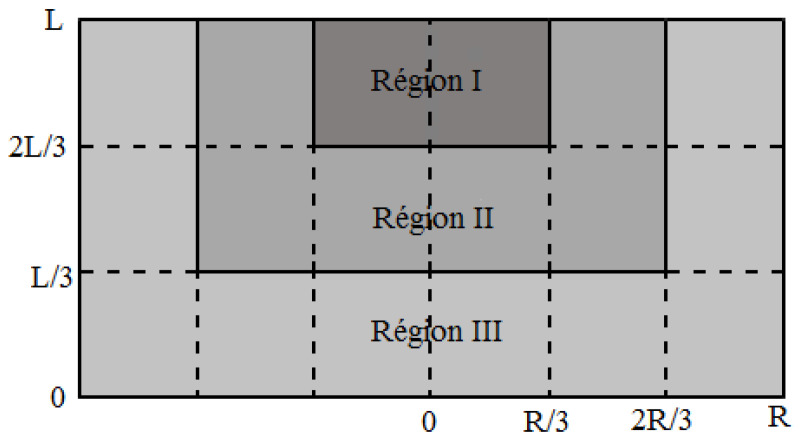
Model of HTS bulk considering homogeneity.

This method has two main limitations. Firstly, it requires two identical HTS bulks. Secondly, the position of the seeds of the two HTS bulks modifies the results of *B_PM_* and the *J_C_* determination, as shown in Figure 12 and Table 1. In Figure 12, different positions of the seeds in relation to each HTS bulk were tested in the GREEN lab. The results of *B_PM_* (Table 1) were different for each case. This is because of the inhomogeneity of the HTS bulk. The value of *J_C_* is bigger close to the seed and smaller far from the seed. A more complex model of the HTS bulk is necessary to consider its inhomogeneity. We propose a model with three critical current density values across three regions. In Figure 13, the HTS bulk of radius R and length L is decomposed in three regions. Region I is a cylinder with a top seed of radius R/3 and length L/3. Region II is a cylinder of radius 2R/3 and length 2L/3 excluding region I. Region III is the cylindrical HTS bulk excluding region I and II. The critical current in region I (radius is bigger than in region II and much bigger than in region III). This decomposition into three regions allows refining the characterization method by mixing new experimental results and numerical simulations. We expect to improve our characterization method using this new model.

## 5. Conclusions

HTS bulks are very promising for electrical motor applications as super magnets or magnetic shields. HTS bulks are built and sold by industrial companies. Electrical motor prototypes were built and successfully tested by different teams using a shielding effect and HTS permanent magnets. The magnetization and characterization of these HTS bulks are necessary to control their use in electrical motor applications. A new direct *J_C_* determination method is presented based on the measurement of *B_P_*. It is a relatively precise method, while it importantly considers the rise rate and Hall probe thickness. It is a valid method to quickly obtain a rough value of *J_C_* for HTS bulk. However, this method does not determine *J_C_* (*B*); as such, improvements will be necessary. 

## Figures and Tables

**Table 1:** Measurements of *T_P_* and *B_PM_* for the three cases (a), (b), and (c).

	Case (a)	Case (b)	Case (c)
*T_P_* (10^−3^ s)	0.93	1.32	1.4
*B_PM_* (T)	2.57	3.02	3.1

## Data Availability

Not applicable.
